# The Study on the Overall Plasma Electrolytic Oxidation for 6061–7075 Dissimilar Aluminum Alloy Welded Parts Based on the Dielectric Breakdown Theory

**DOI:** 10.3390/ma11010063

**Published:** 2018-01-02

**Authors:** Yanfei Chen, Xiaocun Song, Jixue Zhou, Hongtao Liu, Yuansheng Yang

**Affiliations:** 1 Advanced Materials Institute, Shandong Academy of Sciences, Jinan 250014, China; sunshineyanfei@126.com (Y.C.); zhoujx@sdas.org (J.Z.); hongtaoliu@sdas.org (H.L.); 2 Shandong Engineering Research Center for Lightweight Automobiles Magnesium Alloys, Jinan 250014, China; 3 Shandong Key Laboratory for High Strength Lightweight Metallic Materials (HLM), Jinan 250014, China; 4 Institute of Metal Research Chinese Academy of Sciences, Shenyang 110016, China; ysyang@imr.ac.cn

**Keywords:** overall plasma oxidation, dielectric breakdown, ceramic coating, dissimilar metal welded parts, welded joint

## Abstract

Electrical connection of dissimilar metals will lead to galvanic corrosion. Therefore, overall surface treatment is necessary for the protection of dissimilar metal welded parts. However, serious unbalanced reactions may occur during overall surface treatment, which makes it difficult to prepare integral coating. In this paper, an overall ceramic coating was fabricated by plasma electrolytic oxidation to wrap the 6061–7075 welded part integrally. Moreover, the growth mechanism of the coating on different areas of the welded part was studied based on the dielectric breakdown theory. The reaction sequence of each area during the treatment was verified through specially designed dielectric breakdown tests. The results showed that the high impedance overall of ceramic coating can inhibit the galvanic corrosion of the 6061–7075 welded part effectively.

## 1. Introduction

The mix and match use of strong/weak materials can be applied to prepare functionally graded materials (FGM), and it is also an effective way to achieve lightweight structure [1,2]. However, magnesium alloys connected with aluminum or other lightweight alloys would suffer from serious galvanic corrosion, which limits the application of FGM in aviation and marine engineering [3]. The overall surface protection of dissimilar metal connecting parts can curb the generation and extension of corrosion effectively [4]. Therefore, it is necessary to conduct in-depth research on the surface anti-corrosion technology for collaborative usage of lightweight structural materials. Plasma electrolytic oxidation is one of the most effective corrosion protection techniques for magnesium and aluminum alloys [5]. It can not only improve the decorative properties of the workpiece but can also prepare high impedance or insulated ceramic coating with low cost and low energy consumption [6,7].

However, the physical properties, and chemical and electrochemical activity differences between the materials make it difficult to conduct overall surface treatment for dissimilar metal connected parts. Serious unbalanced reactions would occur during the process, which would reduce the overall corrosion resisting performance significantly [8].

In this paper, 6061 and 7075 aluminum alloys were friction stir welded, and overall plasma oxidation treatment was applied to surface protect the welded parts. Friction stir welding (FSW) is an important solid-phase joining method for the preparation of FGM, especially for light alloys such as magnesium, aluminum, copper, titanium and their alloys. It can prevent the occurrence of embrittlement and weakening phase in welding joints [9]. However, FSW changed the property of welding area, therefore the morphology and composition of the coating on the surface of the welding area and base metals (6061/7075) were different. Based on the dielectric breakdown theory, the growth mechanism of ceramic coating on different areas of 6061–7075 welded part was explored and explained. Based on the experimental results obtained, a possible mechanism for the formation of overall ceramic coating was proposed. Furthermore, the results showed that the corrosion resistance of the 6061–7075 welded part was improved and the potential corrosion difference between base metals and welding area was reduced significantly by overall plasma oxidation.

## 2. Experimental

### 2.1. Materials

The substrate materials used in this investigation were 6061 and 7075 aluminum alloys with the chemical compositions shown in Table 1 and Table 2. The 6061 and 7075 aluminum alloys were cut into cubical sheets with a dimension of 50 × 150 × 4 mm^3^. Then, the 6061 aluminum and 7075 aluminum sheets were butt friction stir welded before overall plasma electrolytic oxidation. All solvents and chemicals used were of a chemically pure grade without any further purification.

### 2.2. Pre-Treatment

Butt surface and surface of 6061 and 7075 aluminum sheets were polished mechanically using progressively finer grades of emery papers and then ultrasonically degreased in acetone before friction stir welding. An X5032 type friction stir welding machine equipped with a right-hand thread stirring head was used to weld the polished dissimilar aluminum sheets [10]. The length and diameter of the stir pin were 3.6 and 4 mm, respectively, and the diameter of the shoulder was 16 mm. The stirring head was tilted 2° in the feeding direction. Stirring speed was set at 950 r/min, and the rotating shoulder was pressed into the substrate metals for 0.2 mm and stayed for 5 s to preheat the base metals. The feeding rate was set at 37.5 mm/min.

The beginning and end of the weld joints were cut from the welded 6061–7075 parts by wire-electrode cutting. Then, all these samples were metallographically grounded using 600–1200 grit silicon carbide papers, rinsed with deionized water, ultrasonically degreased in acetone for 20 min, and finally dried in cold air before overall plasma electrolytic oxidation.

### 2.3. Overall Plasma Electrolytic Oxidation

The overall ceramic coatings on the surface of 6061–7075 welded parts were prepared with a JHMAO-DY-200 type 200 kW DC pulse plasma electrolytic oxidation system, which was manufactured at Xi’an University of Technology. The 6061–7075 welded part was used as anode, and a stainless steel sheet was used as the cathode.

The electrolyte was prepared by dissolving 6 g/L Na_2_SiO_3_, 8 g/L KOH, 8 g/L KF, 0.5 g/L Na_2_WO_4_ and 6 g/L (NaPO_3_)_6_ in deionized water. The electrolyte temperature was controlled automatically at 30 ± 5 °C. The pH value before plasma electrolytic oxidation was in the range of 11–12. The whole process (10 min) was carried out under a constant current density of 30 mA/cm^2^ at 500 Hz with a duty cycle of 10% and the maximum voltage applied was 400 V. The treated samples were rinsed with alcohol in ultrasonic bath for 10 min and dried in cold air [11,12].

### 2.4. Characterization and Testing

The thickness of the coating was measured by a Time TT230 type coating thickness gauge (TIME Group INC, BeiJing, China). The surface and cross-sectional morphology of the coatings were investigated by ZEISS EVO MA 10/LS 10 type scanning electron microscope (SEM) (ZEISS Group, Jena, Germany), and their elemental composition was determined by OXFORD X-Max type energy dispersive spectrometer (EDS) (Oxford Instruments, Oxford, UK). To determine the phase composition, a Bruker D2 PHASER X-ray diffraction (XRD) (Bruker Corporation., Karlsruhe, Germany) with Cu Kα radiation was used. The current and voltage of the dielectric breakdown were detected by a Nanjing Changjiang CJ2671S type hipot tester.

**Figure 1:**
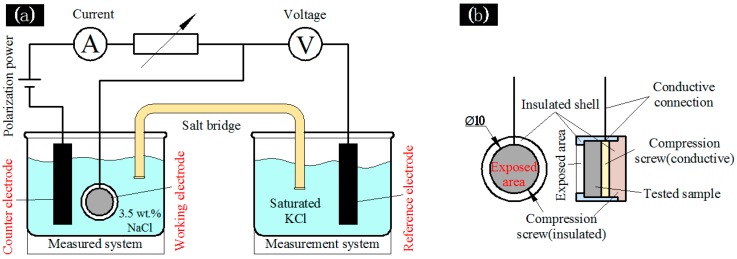
The sealed and insulated fixture for electrochemical test. (**a**) The schematic of three-electrode system; (**b**) the schematic of working electrode [15].

All electrochemical experiments were carried out in a 3.5 wt % NaCl aqueous solution at room temperature using a Shanghai Chenhua CHI660E electrochemical system. The electrochemical cell consisted of a three-electrode system. The tested sample was used as the working electrode. The platinum sheet was used as the counter electrode, and the saturated calomel electrode (Hg/Hg_2_Cl_2_ in saturated solution of KCl) was used as the reference electrode [13]. Specimens for electrochemical test were sampled from the center interface of 6061, 7075 and welding area of the treated dissimilar aluminum connecting parts. The working electrode was set in a custom-made Teflon holder, which had a circular 1 cm window exposed to the electrolyte solution. The exposed circular area was 0.785 cm^2^, as shown in Figure 1. Besides, the scanning speed of the potentiodynamic electrochemical tests was 3 mV/s. The Tafel curves were obtained after the electrodes were placed in the NaCl solution for 60 s, so the electrolytes were fully infiltrated by the solution, and the obtained curves were used to deduce the equilibrium potential *E_corr_* and *i_corr_*. The corrosion resistance of the ceramic layer can be compared by the measured corrosion current intensity *i_corr_* [14].

## 3. Results and Discussion

**Figure 2:**
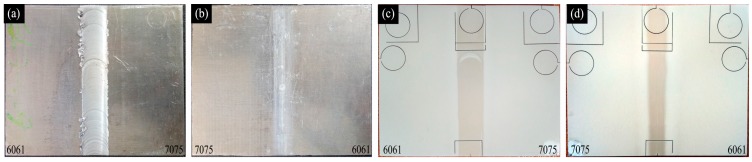
The 6061–7075 welded part: (**a**) top surface; (**b**) bottom surface; (**c**) top surface of treated specimen; and (**d**) bottom surface of treated specimen.

**Figure 3:**
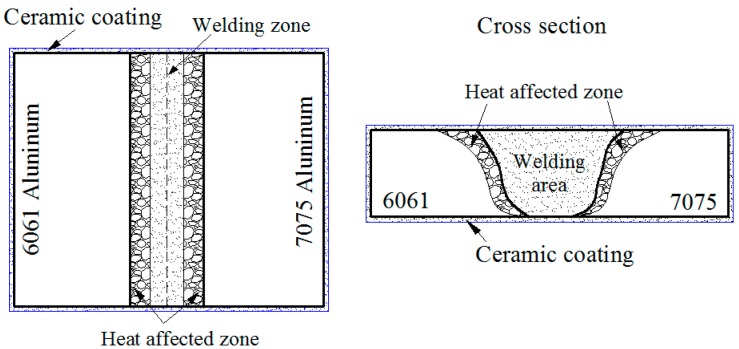
Schematic diagrams of the overall ceramic coating.

The 6061 and 7075 dissimilar aluminum alloys were successfully friction stir welded, as shown in Figure 2a,b. Overall, plasma electrolytic oxidation was applied to surface the to protect the welded parts, and the welded parts were integrally wrapped by a ceramic coating with 10 min overall plasma electrolytic oxidation treatment, as shown in Figure 2c,d. In other words, the ceramic coating grew on all areas contacted with the electrolyte, including 7075, 6061 and the welding joints surface. Thereby, an integral and sealed protective layer formed. As a result, the insulated ceramic coating isolates the 6061–7075 welded part from corrosion media, and broke the electronic circuit of corrosion to improve the corrosion resistance of welded part [16]. The schematic diagram of overall ceramic coating on the surface of 6061–7075 welded parts is presented in Figure 3.

There was a slight color difference between the ceramic coating on base metals (6061/7075, white) and welding area (slight yellow) because the surface elements distribution of welding area was changed by friction stir process. The color of the coating on 6061 and 7075 was almost the same, since the main alloying elements were similar.

**Figure 4:**
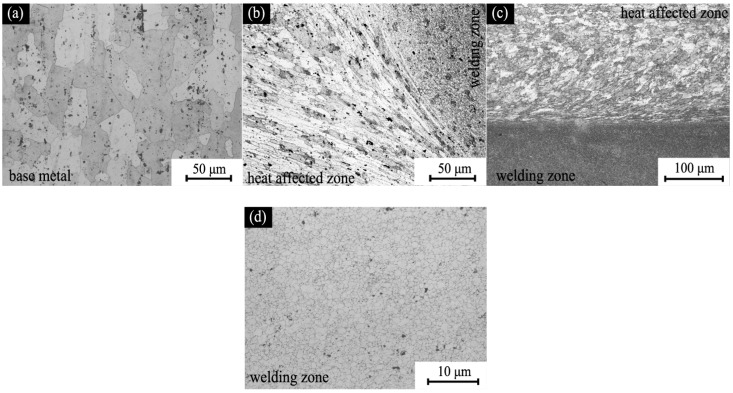
Optical micrographs of welded part: (**a**) 7075; (**b**) cross section of the welding zone; (**c**) top surface of the welding zone; and (**d**) welding zone.

Figure 4 shows the microstructure in different areas of the friction stir welded part. The grain size of the initial base metals was about 40 μm, as shown in Figure 4a; as a similar situation occurred for 6061 aluminum alloy, it is not shown here. The cross section microstructure and the surface microstructure of the welding joint shown in Figure 4b,c revealed significant differences between the welding area and the base metals (heat affected zone). The grain in welding area was broken into extremely small size (about 2 μm) by the stirring pin, as shown in Figure 4d, and the grains in thermo-mechanical affected zone were elongated and deformed. It is obvious that the surface of the welded part was divided into different areas based on the grain type.

### 3.1. SEM Morphology and EDS Analysis of the Ceramic Coating

**Figure 5:**
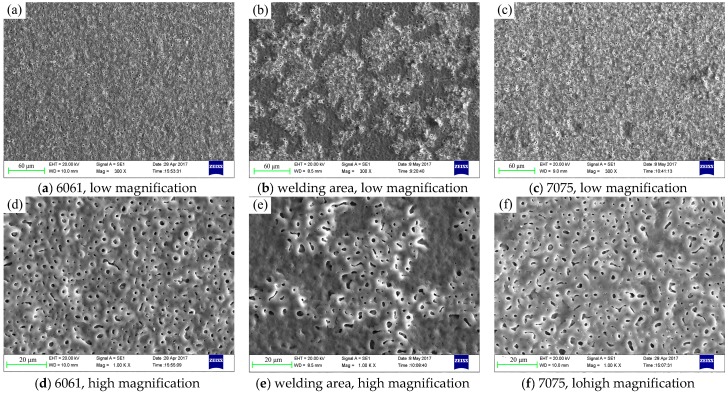
SEM morphology of the ceramic coating on: (**a**,**d**) 6061; (**b**,**e**) welding area; and (**c**,**f**) 7075.

Figure 5 shows the morphology of ceramic coating on the surface of 6061, 7075 and welding area. Considerable amounts of micropores were randomly distributed on the surface of the coatings [17]. Therefore, the morphology of the coating was relatively rough. Micropores formed when the molten oxides and gas bubbles ejected from discharge channels [18,19]. Obviously, the morphology of the ceramic coating on the surface of welding area was different from the coatings on base metals (6061, 7075), as shown in Figure 5a–c. Many island-shaped and volcano-like objects grew on the surface of welding area. Conversely, the coating on 6061 and 7075 surfaces was much more consistent and flat. The observation from high magnification in Figure 5d–f showed that the coatings on 6061 and 7075 had a similar porous structure, and the diameter of the pores was about 0.5–2 μm. However, the island-shaped and porous objects on the coating of welding area seemed to grow on a dense basal layer, and it had a similar porous structure as the coating on 6061/7075, which can be seen in Figure 5d–f. In other words, the ceramic coating on welding area consisted of a porous layer and a dense basal layer from the top to the bottom.

**Figure 6:**
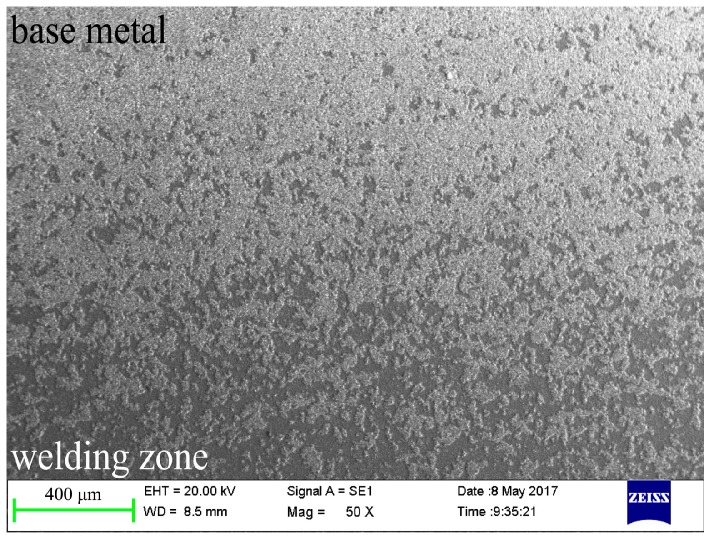
Morphology of the island-shaped porous layer.

**Figure 7:**
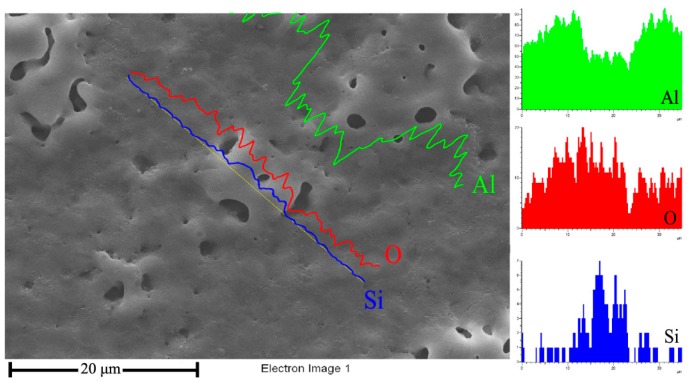
EDS linear scan of the island-shaped porous layer.

Besides, the number of randomly distributed island-shaped porous layers gradually decreased along the 6061/7075 aluminum area to welding area, as shown in Figure 6. To further explore the growth mechanism differences between the ceramic coating on base metals and welding area, EDS linear scan was used to analyze the island-shaped porous layer and the dense basal layer. The results in Figure 7 showed that the intensity of silicon (Si) element rose in the island-shaped porous layer, whereas the intensity of aluminum (Al) decreased.

**Figure 8:**
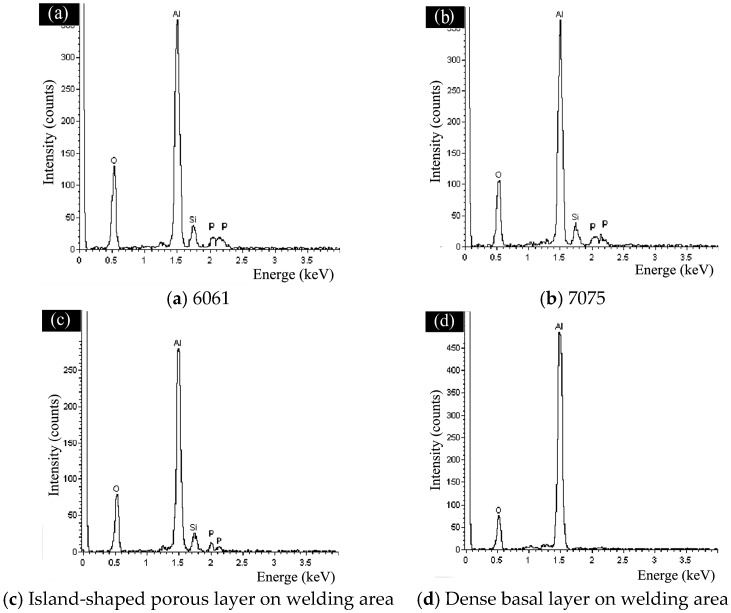
The EDS results of the coating on each areas: (**a**) 6061; (**b**) 7075; (**c**) island-shaped porous layer; and (**d**) dense basal layer.

Moreover, EDS analysis in Figure 8a–d indicated that the porous ceramic coating on 6061/7075 and the porous island-shaped layer on welding area had a similar elemental composition; the intensity of elemental composition was similar too. They consisted of Al, O, Si, and P. However, the dense basal layer on the welding area was made up of O and Al, as shown in Figure 8d, and the main composition was inferred as Al_2_O_3_. The difference reflected that a time sequence or growth priority existed during the growth process of the overall ceramic coating: the dense basal layer formed first on the surface of base metal, and the porous layer then grew on the dense basal layer [15,20,21].

### 3.2. Cross-Section Morphologies and Analysis of the Ceramic Coating

**Figure 9:**
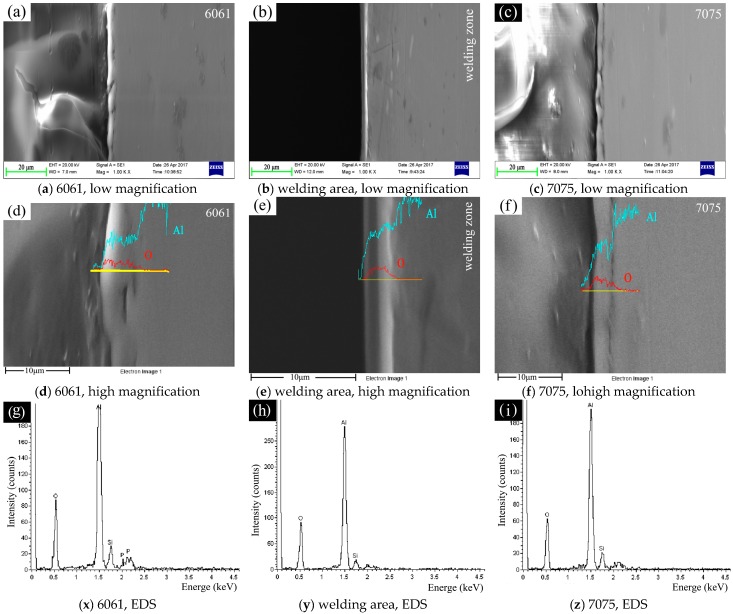
The cross-section morphology and EDS analysis of the ceramic coating on: 6061 (**a**,**d**,**g**); welding area (**b**,**e**,**h**); and 7075 (**c**,**f**,**i**).

Cross-sectional morphology and EDS analysis of the ceramic coatings on the surface of 6061, 7075 and welding area are shown in Figure 9. The thickness of the coating on 6061, 7075 and welding area was about 5, 4 and 3.5 μm on average, respectively. Clearly, the coating on 6061, 7075 and welding area was integrated firmly with the metal substrate by sintered interlocking. In addition, the difference in thickness proved that the reaction priority or sequence of different areas in 6061–7075 welded part existed during overall plasma oxidation.

Besides, the surface morphology in Figure 5 showed that countless micropores distributed on the surface of ceramic coating, while pores were not found in the cross-section morphology in Figure 9d–f, except for a dense layer. This was important evidence that the dense layer occupied a high proportion (close to 100%) of the entire ceramic coating. In fact, the pores would permit more corrosive inter medium to be absorbed into the ceramic coating and decrease the corrosion resistance. In conventional plasma oxidation treatment [22,23], this dense layer was the main protective layer of the ceramic coating, which only accounting for about 60% of the total thickness. Therefore, the current overall plasma oxidation treated 6061–7075 welded parts exhibited excellent integral corrosion resistance, as discussed in Section 3.5.

EDS analysis of the cross section is shown in Figure 9g–i. It was obvious that main constituent elements of the ceramic coating in different area of the 6061–7075 welded part were almost the same. The main elements were Al, O, Si and P, and the intensity of the elements in each area was slightly different.

### 3.3. XRD Analysis of the Ceramic Coating in Each Area of the 6061–7075 Welded Part

**Figure 10:**
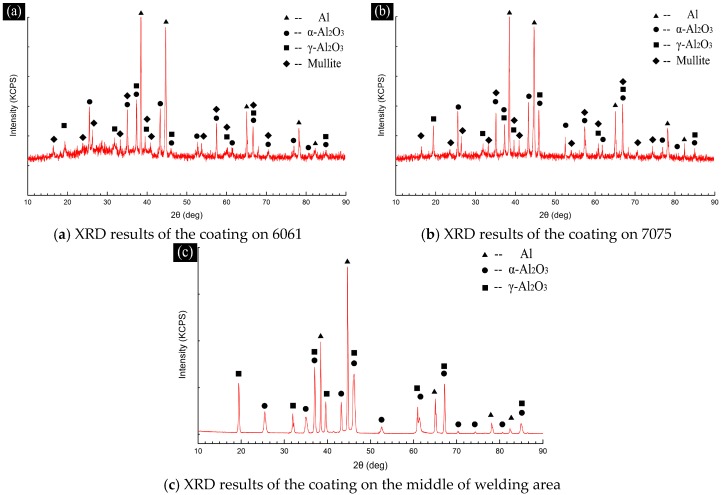
XRD results of the coating: (**a**) 6061; (**b**) 7075; and (**c**) middle of welding area.

X-ray diffraction (XRD) was applied to accurately detect the components of the ceramic coating in different areas of 6061–7075 welded part, and the results are shown in Figure 10.

By comparing the XRD results, the components of the porous coating on the surface of 6061 and 7075 were almost the same, i.e., α-Al_2_O_3_, γ-Al_2_O_3_ and small amount of mullite (3Al_2_O_3_-2SiO_2_). However, the main components of the coating on weld zone (middle position) were α-Al_2_O_3_ and γ-Al_2_O_3_ since the middle position of welding area was mainly covered by dense basal layer and small amount of distributed island-shaped porous layer, as shown in Figure 5b,e, thus the diffraction peak data obtained were mainly those of the dense basal layer. The XRD results were identical to the EDS analysis results in Figure 8 [24].

However, peak intensity of the γ-Al_2_O_3_ decreased from welding area to 7075 and to 6061, while the peak intensity of α-Al_2_O_3_ showed an opposite tendency. This trend was related to the conversion of γ-Al_2_O_3_ to α-Al_2_O_3_ during plasma oxidation process, on the one hand, and the reaction priority and sequence of the different areas in 6061–7075 welded part, on the other hand. The γ-Al_2_O_3_ would convert into α-Al_2_O_3_ irreversibly at 1050–1500 °C and the micro arc generated during plasma oxidation process provided the temperature conditions for the conversion [25].

Besides, there were obvious diffuse scattering peaks in the XRD results of the unpolished porous ceramic coating, as shown in Figure 10a,b, while similar diffuse scattering peaks did not appear in the XRD results of dense basal layer, as shown in Figure 10c. There was some amount of P-containing amorphous or amorphous SiO_2_ in the porous ceramic coating, considering that P was detected in the porous coating by EDS analysis, while crystalline material of P was not found in XRD results [26,27]. According to the growth mechanism proposed in this paper, small amount of SiO_3_^2−^ and PO_4_^3−^ in electrolyte participated in the reaction and formed high temperature melt. The outer layer formed at high temperature melts and sharply cooled under the chilling effect of the electrolyte, during which a certain amount of Si or P-containing amorphous phase formed in the outer shell of the melt. Then, they randomly distributed in the ceramic coating.

In addition, with the temperature conditions produced by the micro arc at about 1470 °C, solid-state thermal reaction occurred between the Al_2_O_3_ and randomly distributed SiO_2_ which resulted in the formation of mullite at the interface of Al_2_O_3_ and SiO_2_ [28], which was part of the growth mechanism of the ceramic coating.

### 3.4. The Growth Mechanism of the Overall Ceramic Coating Based on the Dielectric Breakdown Theory

According to the classic plasma oxidation mechanism [29], the aluminum was rapidly oxidized and ejected from the discharge channels with the generation of discharge sparks. Meanwhile, small amount of SiO_3_^2−^ and PO_4_^3−^ in the electrolyte diffused into the discharge channels to participate in the reaction forming high temperature melt. The high temperature melt then cooled down and formed the dense basal layer. Thus, it was clear that the dense layer, which formed first, was mainly made of aluminum oxide and a small amount of Si and P containing substances, which was verified through EDS and XRD in the previous sections. As the plasma oxidation proceeded, the ceramic coating was much more difficult to breakdown, since it was thickening constantly. As a result, the number of discharge channels decreased, and the oxidation of the aluminum weakened. The SiO_3_^2−^ and PO_4_^3−^ in electrolyte began to participate in the reaction massively and the formed particles deposited on the surface of the initial formed dense layer. Therefore, the content of Si and P in this layer was higher than that of the initial dense layer [30,31].

**Figure 11:**
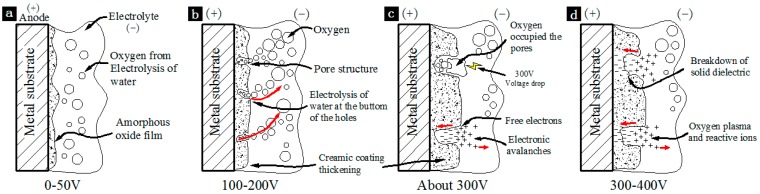
The diagram of dielectric breakdown at the voltage of (**a**) 0–50 V; (**b**) 50–100 V; (**c**) about 300 V; (**d**) 300–400 V.

Obviously, the breakdown of dielectric was the key to the growth mechanism of the overall ceramic coating in both the arcing and the breakdown of ceramic coating. Figure 11 shows a diagram of dielectric breakdown process. At 0–50 V low voltage stage (Figure 11a), aluminum substrate reacted with the alkaline electrolyte, a layer of colloidal Al(OH)_3_ formed on the surface of aluminum substrate, which caused the most primitive colloidal dielectric breakdown behavior. The aluminum substrate started to be oxidized as a result and a layer of amorphous Al_2_O_3_ with certain amount of resistance formed gradually. At 100–200 V voltage stage (Figure 11b), the Al_2_O_3_ film thickened gradually and the pore structure formed around the discharge channels. Accordingly, the electrical breakdown and the electrolysis of water occurred at the bottom of the holes. When the voltage rose to about 300 V (Figure 11c), the electrolysis of water was extremely intense so that the entire pore was occupied by the produced oxygen (O_2_). Oxygen bubble was ionized at a voltage drop of about 300 V, and free electrons were generated which triggered the “electronic avalanches” (free electron impact ionization, the number of free electrons was doubled by collisions to 2^n^, where “n” is the number of collisions) [32]. Meanwhile, the corona discharge occurred when the oxygen plasma and reactive ions began to form. As a result, the oxide coating on the bottom of the holes broke down and the plasma oxidation entered the spark discharge stage with the ceramic coating thickening rapidly.

**Figure 12:**
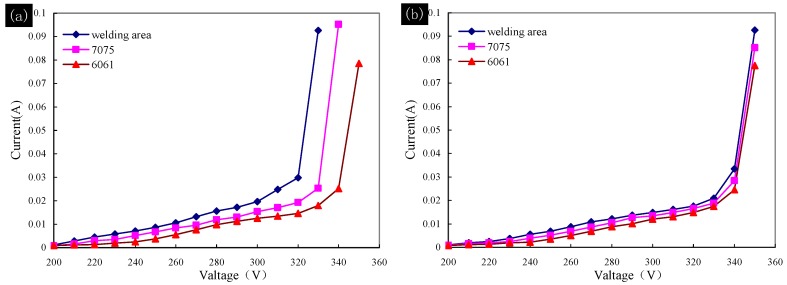
The breakdown tests of the samples from each area of the welded part: (**a**) samples from treated welded part; and (**b**) samples from untreated welded part and then plasma oxidation treated, respectively.

However, it was different for the overall plasma oxidation of the 6061–7075 dissimilar aluminum welded part. Since a chemical/electrochemical activity difference exists between the different areas of dissimilar metal welded part, there is a reaction priority or sequence in the plasma oxidation process. Thus, the breakdown voltage of the solid-state dielectric formed on each area of the welded part was different. To verify the reaction sequence, two breakdown tests were carried out. The first test (a) sampled from an overall plasma oxidation treated welded part with a treating time of 60 s. The second test (b) sampled from untreated welded part. The untreated 6061, 7075 and welding area samples were then plasma oxidation treated for 60 s under the same experimental conditions. The breakdown tests results are shown in Figure 12.

The results in Figure 12 show an interesting law. The 6061, 7075 and welding area samples in test (b) were taken from an untreated welded part, and a coating with similar thickness would grow on surface of the samples after 60 s plasma oxidation. The tests results in Figure 12b thusly show a similar breakdown voltage of dielectric at about 340 V. However, the breakdown voltage of samples in test (a) was different from each other, and the samples were taken from different area of overall treated welded part. The breakdown voltage of the 6061, 7075 and welding area samples in test (a) was about 340, 330 and 320 V, respectively. This was because the thickness of the ceramic coating on each area of overall treated welded part was different. The coating on welding area was the thinnest, as shown in Figure 9, so it was the easiest area to breakdown. The difference reflected the existence of reaction priority and sequence. It could be summarized as the ceramic coating formed preferentially on the surface of 6061 aluminum, followed by 7075 aluminum and then the welding area, although the ceramic coating on each area grew at the same time.

The growth mechanism of the overall ceramic coating and the phenomenon observed could be well explained based on the dielectric breakdown theory. The reaction priority and sequence were related to the physical and chemical properties of the 6061, 7075 and the welding area. The conductive performance of 6061 was better than that of 7075 and much better than welding area, because the reinforcing phase would reduce the conductivity [33]. Therefore, the current passed through the 6061 preferentially during the overall plasma oxidation of the 6061–7075 welded part. The alkaline dielectric on the surface of 6061 broke down first along with the coating growing on the surface of 6061, and solid-state dielectric further accelerated the formation of the coating on 6061. Then, the high impedance ceramic coating changed the surface conductivity of 6061, and the dielectric on surface of 7075 was easier to breakdown than that of 6061 in this stage. The same mechanism occurred in the welding area which caused the reaction priority and sequence observed.

### 3.5. Corrosion Resistance of Ceramic Coating in Different Areas of the Welded Part

Corrosion potential difference exists among different kinds of metals, and the conductive contact of dissimilar metals will cause serious galvanic corrosion [34]. As a result, the corrosion rate of the metal with lower corrosion potential (act as anode) increased and the metal with higher corrosion potential (act as cathode) was the opposite [35]. Accordingly, it would significantly reduce the service life of the dissimilar metal welded/connected parts. The overall micro arc oxidation could improve the corrosion resistance of each material as well as reduce the corrosion potential difference between different metals i [15,36]. Furthermore, the insulated integral ceramic coating isolated the 6061–7075 welded parts from corrosion media and broke the electronic circuit of corrosion, which meant the generation and extension of the corrosion were curbed effectively and it was the key technology to reduce the galvanic corrosion for lightweight alloys [15].

The corrosion resistance of 6061 aluminum alloy was higher than that of 7075, since the main alloying elements of the 7075 were Zn and Mg (Table 2). The main intermetallic compounds of 7075 were MgZn_2_ and Mg_2_Si [37]. However, MgZn_2_ and Mg_2_Si would dissolve and precipitate to Mg and Zn containing precipitation phases when corrosion occurred [38], which made 7075 exhibit poor corrosion resistance. On the contrary, the main intermetallic compounds of 6061 were Mg_2_Si, which is more stable than MgZn_2,_ resulting in better corrosion resistance [39,40,41]. The corrosion potential (*E_corr_*) and corrosion current density (*I_corr_*) of 6061 and 7075 are shown in Table 3. The *E_corr_* of 6061 and 7075 was −0.7655 and −0.7999 V, respectively, which verified the analysis above.

**Figure 13:**
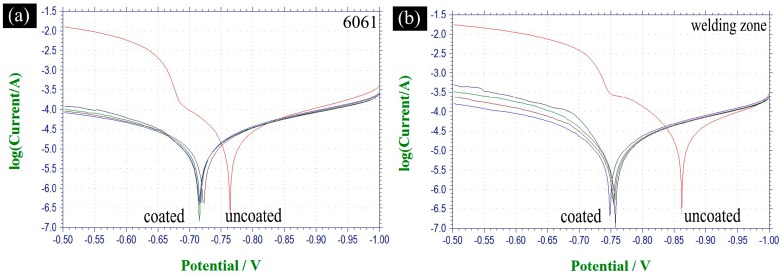

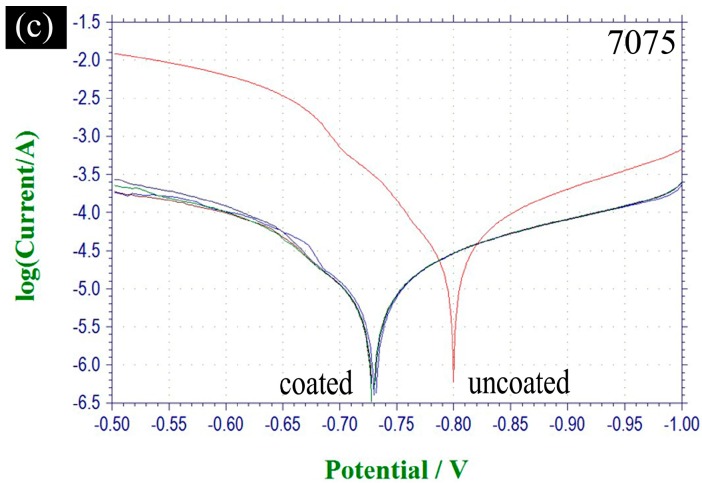
Polarization curves of: (**a**) 6061; (**b**) welding area; and (**c**) 7075.

Electrochemical polarization curves of the coated/uncoated base metals (6061/7075) and welding area of the 6061–7075 dissimilar aluminum alloys friction stir welded parts are shown in Figure 13. The *E_corr_* and *I_corr_* were extracted from the potentiodynamic polarization curves via Tafel region extrapolation and the parameters summarized in Table 3. It was clear that the corrosion resistance of 6061/7075 aluminum substrate and welding area were enhanced with the overall plasma oxidation treatment. The *E_corr_* of 6061 was improved from −0.7665 to −0.7172 V (improved 8.9%), and the *I_corr_* decreased from 4.359 to 2.138 e^−5^ A/cm^2^ (decreased 50.9%). The *E_corr_* of 7075 was improved from −0.7999 to −0.7289 V (improved 8.8%) and the *I_corr_* was decreased from 5.183 to 1.636 e^−5^ A/cm^2^ (decreased 68.4%). Similarly, the *E_corr_* of the welding area was increased to −0.7537 from −0.8633 V (improved 12.7%) and the *I_corr_* was decreased to 1.595 from 4.898 e^−5^ A/cm^2^ (decreased 67.4%). All the electrochemical test results showed that the corrosion resistance of each area was improved.

Besides, the overall plasma oxidation treatment reduced the potential corrosion difference between each area. The *E_corr_* of the welding area was lower than the *E_corr_* of 6061/7075 aluminum, which means that the friction stir welding reduced the corrosion resistance of the welding joint. As a result, the potential galvanic corrosion would occur first on the welding joint. However, the corrosion potential difference between 6061 and 7075 was 0.0344 V before overall plasma oxidation treatment, and it decreased to 0.0126 V with the overall treatment. Moreover, the *E_corr_* difference between 6061 and welding area decreased from 0.0978 to 0.0365 V, and the *E_corr_* difference of 7075 and welding area decreased from 0.0634 to 0.0239 V. All the data indicate that corrosion potential difference between different areas was reduced which meant better galvanic corrosion resistance and overall protective performance.

## 4. Conclusions

(1) The 6061–7075 dissimilar aluminum welded part was successfully treated by overall plasma oxidation and the welded part was integrally wrapped by a ceramic coating.
(2) The ceramic coating on the surface of welding area was different from the coating on base metal (6061/7075) in both morphology and composition. The coating on the welding area was mainly made of α-Al_2_O_3_ and γ-Al_2_O_3_, while the coating on the 6061 and 7075 consisted of α-Al_2_O_3_, γ-Al_2_O_3_, mullite and small amount of P-containing amorphous.
(3) Reaction priority and sequence during overall plasma oxidation of 6061–7075 welded part was observed. The plasma oxidation reaction prefers to occur in the sequence of 6061 aluminum, 7075 aluminum, and welding area.
(4) The overall plasma oxidation improved the corrosion resistance of all areas on 6061–7075 welded part and reduced the corrosion potential difference between different areas.

## Figures and Tables

**Table 1:** Chemical composition of 6061 aluminum alloy.

Si	Fe	Cu	Mn	Mg	Cr	Zn	Al
0.59	0.42	0.19	0.01	0.93	0.18	0.12	balance

**Table 2:** Chemical composition of 7075 aluminum alloy.

Si	Fe	Cu	Mn	Mg	Cr	Zn	Al
0.05	0.16	1.43	0.02	2.58	0.23	5.79	balance

**Table 3:** The *E_corr_* and *I_corr_* of the uncoated/coated 6061, 7075 and welding area.

	Area	6061		Welding Area		7075	
Treatment		*E_corr_*/V	*I_corr_/*e^−5^ A/cm^2^	*E_corr_*/V	*I_corr_/*e^−5^ A/cm^2^	*E_corr_*/V	*I_corr_/*e^−5^ A/cm^2^
Uncoated		−0.7655	4.359	−0.8633	6.239	−0.7999	5.183
Coated		−0.7172	2.138	−0.7537	2.032	−0.7289	1.636
